# The impact of selcopintide on periodontal regeneration in a class II furcation defect model: a radiographic and histomorphometric analysis

**DOI:** 10.1007/s00784-025-06334-0

**Published:** 2025-04-24

**Authors:** Jungwoo Jung, Jungwon Lee, Seunghee Lee, Ki-Tae Koo, Yang-Jo Seol, Joo-Cheol Park, Jeongmin Park, Su-Jin Park, Chul Son, Seong Min Roh, Jin-Seok Seo, Yong-Moo Lee

**Affiliations:** 1 Department of Periodontology and Dental Research Institute, School of Dentistry, Seoul National University, 101, Daehak-ro, Jongno-gu, Seoul, 03080 Republic of Korea; 2 One-Stop Specialty Center, Seoul National University Dental Hospital, Seoul, Republic of Korea; 3 Laboratory for the Study of Regenerative Dental Medicine, Department of Oral Histology and Developmental Biology, School of Dentistry, Seoul National University, Seoul, Korea; 4 Regenerative Dental Medicine R and D Center, HysensBio Co., Ltd, 10 Dwitgol-ro, Gwacheon-si, Gyeonggi-do Korea

**Keywords:** Biomaterials, CPNE7 protein, Peptides, Furcation defects, Guided tissue regeneration, Animal experimentation

## Abstract

**Objectives:**

This study investigated the effect of Selcopintide (SCPT) on periodontal regeneration using surgically created class II furcation defects in an animal model, employing both radiographical and histological evaluations.

**Materials and methods:**

Class II furcation defects were surgically induced in six beagle dogs. Each defect was unilaterally and randomly assigned to one of five treatment groups: (1) no treatment (control), (2) guided tissue regeneration (GTR) with a collagenated bone substitute and a collagen membrane, (3) GTR with an enamel matrix derivative (GTR/EMD), (4) GTR with 2 mg/ml SCPT (GTR/SCPT2), or (5) GTR with 4 mg/ml SCPT (GTR/SCPT4). Six weeks after initial surgery, identical procedures were performed on the contralateral area. Healing outcomes were assessed at 6 and 12 weeks through radiographic and histomorphometric analyses. The primary outcome was the length of the root surface covered by new cementum assessed histologically and the secondary outcome included other histomorphometric and radiographic healing outcomes.

**Results:**

Radiographically, the GTR/SCPT groups showed significantly greater new bone volume than the control group at both 6- and 12-week timepoints (*p* < 0.05). Histologically, specimens treated with both formulations of SCPT displayed better-organized periodontal ligaments and improved cementum formation, similar to those treated with EMD. The group with the highest concentration of SCPT (GTR/SCPT4) exhibited the greatest amount of new cementum formation.

**Conclusions:**

Within the limitations of this study, both formulations of SCPT demonstrated significant regenerative potential in periodontal tissues, with outcomes comparable to those of EMD treatment, suggesting SCPT’s viability as an alternative bioactive agent for periodontal regeneration in class II furcation defects.

**Clinical relevance:**

This study suggests that SCPT could serve as a viable alternative bioactive agent for periodontal regeneration in class II furcation defect in clinical settings.

## Introduction

Periodontal regeneration, which involves the formation of new cementum, alveolar bone, and functional periodontal ligament on a root surface previously affected by periodontal disease, represents the ultimate goal of periodontal treatment [1]. Over the past few decades, extensive research has focused on achieving comprehensive periodontal tissue regeneration in conditions such as gingival recession, furcation defects, and intraosseous defects [2–4]. Recent guidelines have made evidence-based recommendations on the use of periodontal regenerative surgery in deep intrabony defects and class II furcation defects (especially in the mandible), to improve outcomes in terms of attachment gain and pocket depth reduction [5]. However, despite the development of various regenerative techniques, achieving predictable outcomes remains a challenge due to the associated complications and the high level of expertise required [6, 7]. Furthermore, histological studies of regenerative treatments often reveal the formation of a long junctional epithelium, indicating a tendency towards repair rather than true regeneration [8, 9].

The principle of guided tissue regeneration (GTR) employs a barrier to prevent the migration of epithelial and connective tissue cells into the defect area, while selectively encouraging the repopulation by periodontal ligament cells and mesenchymal cells [10]. Over the past 40 years, efforts to improve GTR outcomes have focused on testing various materials and bioactive agents, including bone substitutes, barrier membranes, enamel matrix derivative (EMD), platelet-derived growth factor (PDGF), fibroblast growth factor, bone morphogenetic protein (BMP), and hyaluronic acid [11–15]. These bioactive substances are often combined with bone grafting materials to maintain space and prevent rapid resorption, thus prolonging their effects [16, 17]. In terms of its regenerative potential, EMD has been accepted as one of the most well-established regenerative agents, and several pre-clinical studies have verified the histological outcomes of other bioactive substances in treatment of periodontal defects as well [5, 18, 19].

Recently, extensive efforts to further explore novel bioactive agents have been made and one promising candidate is copine-7 (CPNE7). CPNE7, a member of the copine protein family, is known to interact with various cell signaling systems and cytoskeletal proteins. Copines, characterized by two C2 domains, bind to phospholipid membranes in the presence of calcium ions [20, 21]. CPNE7, a dental epithelium-derived protein secreted by pre-ameloblasts, has attracted attention in dentin regeneration research due to its role in stimulating odontoblast differentiation [22–24]. Recent in vitro studies have reported that peptides derived from CPNE7 exhibit osteogenic potential when applied to mesenchymal stem cells [25]. Previous studies have also demonstrated CPNE7’s efficacy in regenerating periodontal tissue in animal models using tooth implantation or alveolar bone defects [26–29].

The synthetic peptide selcopintide (SCPT), derived from CPNE7, corresponds to a 10-amino-acid sequence (KYKQKRRSYK) found in the hCPNE7 protein [25]. Previous research has demonstrated that SCPT enhances the recovery of the periodontal ligament by promoting the attachment of periodontal ligament cells. This effect is mediated through TAU-driven cytoskeletal reorganization and the regulation of cementum attachment protein (CAP) in three-wall defect models [30]. Although ongoing studies are exploring the potential of SCPT for periodontal tissue regeneration, research focusing on its effects in furcation defect models—particularly challenging scenarios for periodontal regeneration—is still scarce. This lack of data highlights a significant opportunity to further explore SCPT’s capabilities in complex periodontal regeneration contexts.

The objective of this study is to explore the impact of SCPT on periodontal regeneration using surgically created class II furcation defects in an animal model, employing both radiological and histological assessments.

## Materials and methods

### Animals

This study received approval from the Seoul National University Animal Experiment Ethics Committee (SNU-230410-2-1). We used six male beagle dogs, each approximately 1 year old and weighing between 10 and 12 kg, for the experiment. All experimental procedures were conducted in accordance with the ARRIVE 2.0 guidelines (Animal Research: Reporting of In Vivo Experiments) [31]. The dogs were individually housed in cages measuring 900 mm in width, 800 mm in depth, and 800 mm in height. We maintained environmental conditions at a constant temperature of 23 ± 2 °C and a relative humidity of 50 ± 10%. The facility enforced a 12-hour light/dark cycle and provided between 12 and 18 air changes per hour.

### Sample size calculation

The sample size calculation was based on a previous study by Bai et al. (2022), which examined CPNE7 in a periodontal defect model [30]. For this study, we considered a 13% difference between the experimental and control groups to be statistically significant, assuming a standard deviation of 7% (effect size d = 1.857) for the new cementum length. The sample size calculation was conducted using G*Power software (Heinrich Heine University, Düsseldorf, Germany). Setting the alpha at 0.05 and aiming for a power of 80%, the analysis determined that a minimum of six beagle dogs would be necessary to confirm superiority between the groups.

### Experimental design

**Fig. 1:**
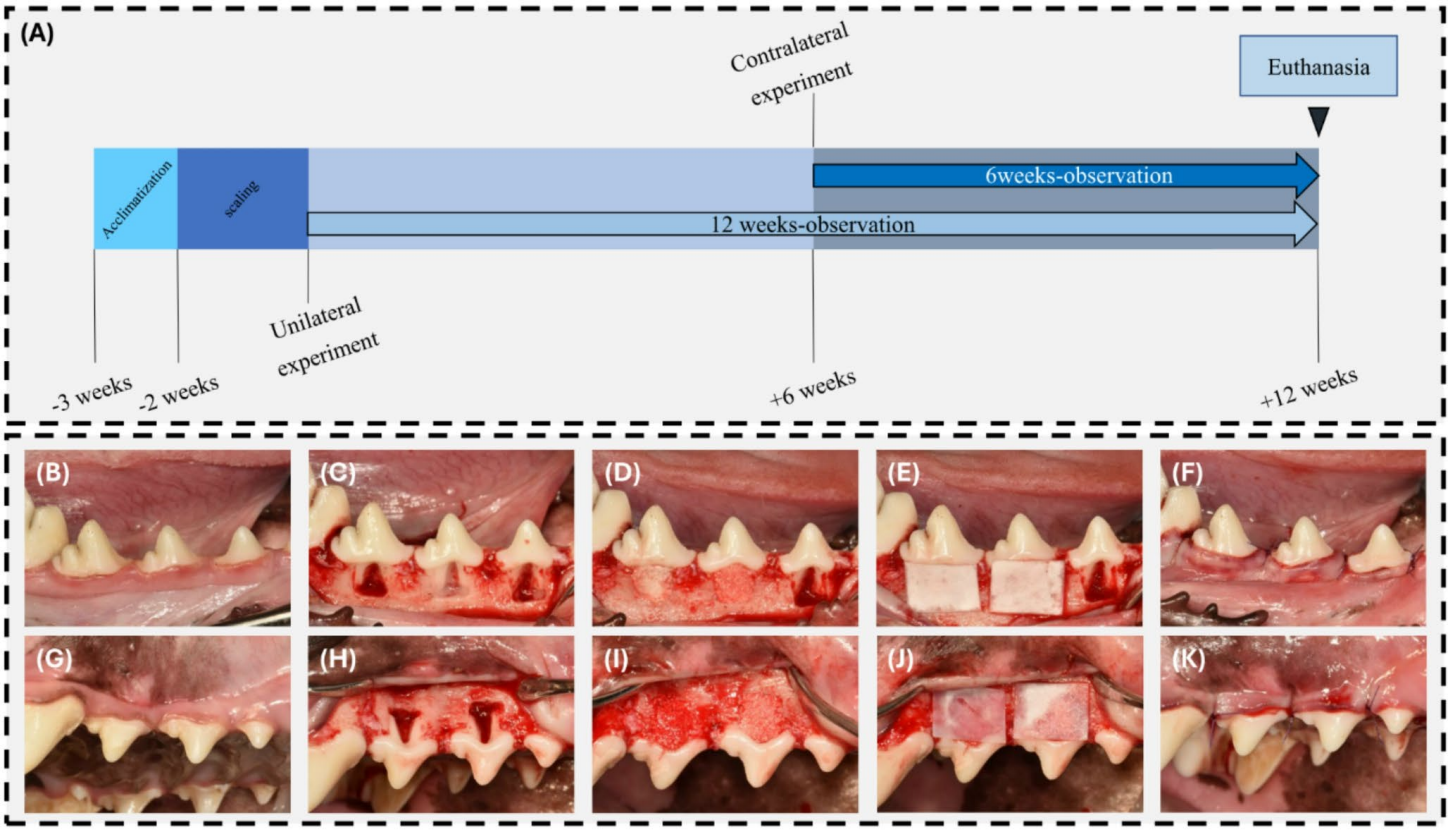
Experimental timeline and surgical procedures for furcation defect treatment. (**A**) Schematic of the experimental design. After a 1-week acclimatization period, scaling was conducted 2 weeks before surgery, and a unilateral furcation defect was created and treated. After 6 weeks, defects were created and treated in the premolar area of the contralateral side using the same method. The animal was sacrificed at 12 weeks after the first surgery. (**B**-**K**) Surgical procedures for each group. (**B**) Preoperative view of the mandible. (**C**) Creation of periodontal defects with 5 mm height and 5 mm width around the teeth. (**D**) Treatment was administered according to the group assignment (PM2: control, PM3: GTR, PM4: GTR/EMD). (**E**) Application of collagen membrane on the defect. (**F**) Sutures were performed at the surgical sites. (**G**) Preoperative view of the maxilla. (**H**) Creation of periodontal defects around the teeth. (**I**) Treatment was administered according to the group assignment (PM3: GTR/SCPT2, PM4: GTR/SCPT4) (**J**) Application of collagen membrane on the defect. (**K**) Sutures were performed at the surgical sites

The timeline of the experiment is described in Fig. 1A. 3 weeks prior to surgery, the animals were given an acclimatization period of one week. Subsequently, scaling was done on the animals 2 weeks prior to surgery. On the day of surgery, class II furcation defects were surgically induced unilaterally on three mandibular premolars and two maxillary premolars of each beagle dog. Within one animal, the five treatment groups were randomly allocated to each furcation defect without redundancy, creating a 12-week observation group. Simple randomization was done with an Excel random number table. The five treatment groups include:

- Control: No treatment.
- GTR: GTR was performed using a collagenated block-type bone substitute (OSTEON Xeno Collagen, Genoss, Gyeonggi-do, Republic of Korea) along with a collagen barrier membrane (Collagen Membrane-P, Genoss, Gyeonggi-do, Republic of Korea).
- GTR/EMD: After root conditioning with EMD (Emdogain, Straumann, Basel, Switzerland), GTR was performed using a collagenated block-type bone substitute soaked in EMD for 10 min along with a collagen barrier membrane.
- GTR/SCPT2: After root conditioning with SCPT (2 mg/ml), GTR was performed using a collagenated block-type bone substitute soaked in SCPT (2 mg/ml) for 10 min along with a collagen barrier membrane.
- GTR/SCPT4: After root conditioning with SCPT (4 mg/ml), GTR was performed using a collagenated block-type bone substitute soaked in SCPT (4 mg/ml) for 10 min along with a collagen barrier membrane.

Six weeks after the initial surgery, the same procedure was repeated on the contralateral maxillary and mandibular premolars, creating a 6-week observation group. Twelve weeks after the first surgery, the animals were euthanized through an intravenous overdose of potassium chloride.

### Surgical procedure

The surgical procedure involved inducing general anesthesia through an intravenous administration of a combination of tiletamine/zolazepam (0.1 mg/kg, Zoletil, Virbac, Carros, France), xylazine (2.3 mg/kg, Rompun, Bayer Korea, Ansan, Korea), and atropine sulfate (0.05 mg/kg, Jeil, Daegu, Korea). The surgical site was then disinfected with an iodine solution. To minimize pain and bleeding, local anesthesia was administered using lidocaine with epinephrine at a concentration of 1:100,000 (Huons Co. Ltd., Seongnam, Korea).

The experiment involved the unilateral mandibular second, third, and fourth premolars, as well as the maxillary third and fourth premolars of six adult dogs (Fig. 1B, G). A full-thickness flap was raised following an intrasulcular incision that extended one tooth beyond the experimental area. Class II furcation defects were created using a dental round bur with saline irrigation. These defects were standardized to a rectangular shape, each measuring 5 mm in width and height, with the depth reaching beyond the center of the bifurcation area. The exposed root surfaces were meticulously debrided using Gracey curettes to eliminate all remaining periodontal ligament and cementum. Root surface conditioning was then performed using a 24% EDTA gel (Prefgel, Straumann, Basel, Switzerland) for 2 min. A reference notch was created on the most apical root surface of each defect using a #1 round bur (Fig. 1C, H). Five furcation defects were created in the unilateral mandible and maxilla, and randomly assigned to one of five treatment groups without redundancy (Fig. 1D, E,I, J). The flaps were then repositioned and sutured using 5 − 0 Monosyn (B. Braun, Melsungen, Germany) with interrupted sutures (Fig. 1F, K). Postoperatively, antibiotics (cefazolin, 20 mg/kg) and analgesics (tramadol HCl, 4 mg/kg) were administered intravenously. Sutures were removed one week post-surgery. All surgical procedures were carried out by a single experienced surgeon (J.J.) to avoid procedure differences between operators.

### Radiological analysis

Radiological examinations of the specimens were performed using a micro-computed tomography device (SkyScan-1173, Kontich, Belgium). The resulting two-dimensional images were saved as 2240 × 2240 pixel BMP files. Subsequently, these images were reconstructed into three-dimensional representations using CTVox software (Bruker, Kontich, Belgium).

Volumetric analysis of the defects was conducted using CTAn software (Bruker, Kontich, Belgium). The volume of interest (VOI) was defined as the region of interest (ROI) extending from above the reference groove to below the root furcation in the sagittal plane. Analysis of the volume was performed in interpolated mode from the lingual to the buccal direction. The lingual boundary was established at the center of the furcation area, with the location of the root canal serving as a reference point. The buccal boundary was determined by the contour of the adjacent alveolar bone.

An 8-bit grayscale threshold was used to identify new bone formation within the VOI. The grayscale range for new bone was optimized through iterative adjustments and ultimately set between 45 and 69 [18]. The results were expressed as the percentage of new bone volume relative to the total volume (NBV) within the VOI. NBV was measured using micro-CT at 6 and 12 weeks, respectively.

### Histological Preparation

Tissues excised from the maxilla and mandible were fixed in a 10% formaldehyde solution for two weeks. Following this, the specimens were decalcified using a 12.5% EDTA solution for six months. After decalcification, the tissues were embedded in paraffin wax and sectioned mesiodistally to a thickness of approximately 3 μm, parallel to the long axis of the tooth.

The tissue sections were stained using hematoxylin-eosin and Masson’s trichrome. Histological analysis was conducted with an optical microscope (DP72, Olympus, Tokyo, Japan). Digital micrographs were captured using DP Controller software (Olympus, Tokyo, Japan).

### Histometric analysis

**Fig. 2:**
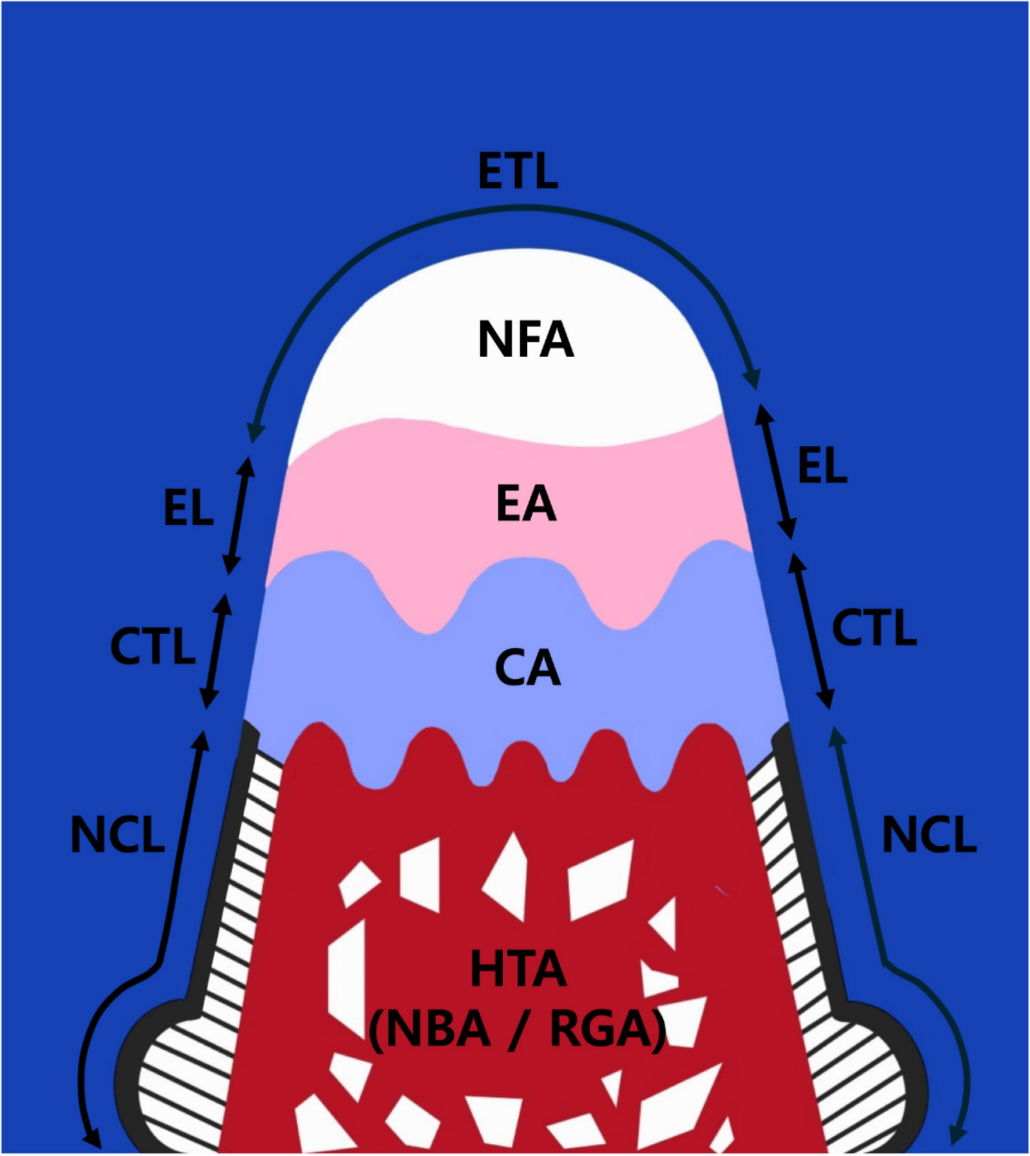
Schematic illustration of histomorphometric analysis of periodontal tissue regeneration ETL: Exposed tooth length EL: Epithelial tissue length CTL: Connective tissue length NCL: New cementum length NFA: Non-filled area EA: Epithelial tissue area NBA: New bone area RGA: Residual graft area CA: Connective tissue area HTA: Hard tissue area

For histometric analysis, a slide representing the most central part of the created defect was selected, ensuring it included a clearly visible reference groove on the lower side. Measurements were conducted twice by a single examiner in 2-week intervals and intra-examiner reliability was confirmed. ImageJ software (National Institutes of Health, Bethesda, MD, USA) was used for the measurements, adhering to parameters established in previous research [32]. Histometric measurements were conducted at 6 and 12 weeks, respectively (Fig. 2).

#### Linear measurements

① Total root length (TRL): total length of the root surface in the furcation defect area, including exposed tooth length, epithelial length, connective tissue length, and new cementum length.

② Exposed tooth length (ETL): length of the root surface not covered by any tissue.

③ Epithelial tissue length (EL): length of the root surface covered by epithelial tissue.

④ Connective tissue length (CTL): length of the root surface covered by connective tissue.

⑤ New cementum length (NCL): length of the root surface covered by new cementum.

#### Area measurements

① Non-filled area (NFA): furcation area not filled by any tissue.

② Epithelial tissue area (EA): furcation area filled with epithelial tissue.

③ New bone area (NBA): furcation area filled with new bone.

④ Residual graft area (RGA): furcation area filled with residual bone graft material.

⑤ Connective tissue area (CA): furcation area filled with connective tissue.

⑥ Hard tissue area (HTA): furcation area filled with new bone and residual bone graft material including NBA and RGA.

### Statistical analysis

Outcome parameters derived from radiological and histomorphometric analyses were calculated and are presented as mean and standard deviation. The statistical analyses were performed by one blinded examiner (J.L.). The Shapiro-Wilk test was performed to determine whether the data fits a normal distribution. Statistically significant differences among five groups were identified using one-way analysis of variance, followed by Tukey’s multiple comparison test when there was a significant difference among the groups. Intra-examiner reliability was assessed using the intraclass correlation coefficient (ICC). ICC of histometric measurement was 0.934, indicating a high level of reliability (*p* < 0.05). The values were analyzed using the GraphPad Prism version 9.4.0 (Graphed Software, San Diego, CA, USA) statistical software. The significance level was set at *p* < 0.05.

## Results

### Clinical observations

All experimental animals remained healthy throughout the study period, with no post-operative complications reported.

### Radiological analysis

**Fig. 3:**
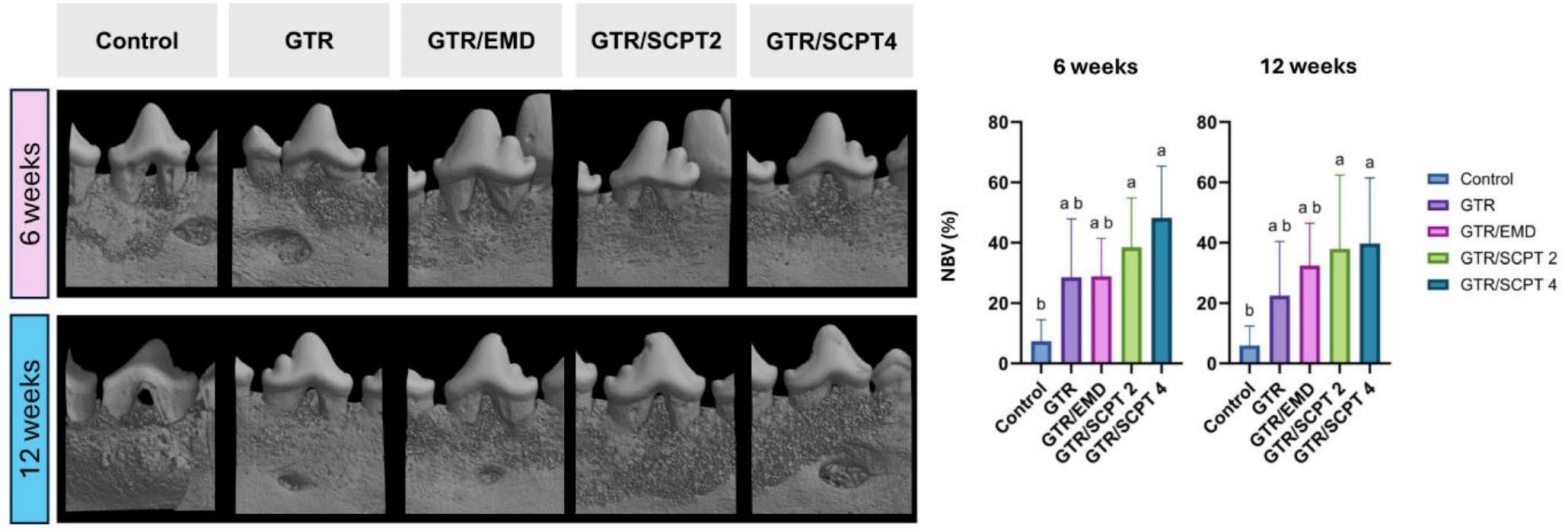
Radiographic analysis of periodontal bone regeneration after various treatments at 6 and 12 weeks. On the left, three-dimensional reconstruction images of periodontal defects are shown at 6 weeks (top row) and 12 weeks (bottom row) for each treatment group: control (no treatment), GTR (guided tissue regeneration), GTR/EMD (GTR with enamel matrix derivative), GTR/SCPT2 (GTR with 2 mg/ml SCPT), and GTR/SCPT 4 (GTR with 4 mg/ml SCPT 4). On the right, bar graphs depict the percentage of new bone volume (NBV) analyzed using micro-CT at 6 and 12 weeks. Different letters, a, and b, indicate statistical significance under Tukey’s multiple comparison test

The results of the micro-CT analysis are presented in Fig. 3. At the 6-week observation point, the percentages of NBV revealed distinct differences among the treatment modalities (*p* < 0.05). The GTR and GTR/EMD groups showed increases in NBV compared to the control group; however, these increases were not statistically significant (28.55 ± 19.33% for GTR group, 28.91 ± 12.53% for GTR/EMD group). In contrast, the GTR/SCPT2 and GTR/SCPT4 groups demonstrated significantly higher NBV than the control group (38.49 ± 16.41% for GTR/SCPT2 group, 48.24 ± 17.20% for GTR/SCPT4 group). It is important to note that increasing the concentration of SCPT did not enhance bone regeneration, as NBV values did not show a significant difference for either concentration (2 mg/ml and 4 mg/ml).

At the 12-week observation, a pattern similar to the 6-week results was noted, showing significant differences among the groups (*p* < 0.05), with the GTR/SCPT2 and GTR/SCPT4 groups maintaining higher NBV levels compared to the control group (37.95 ± 24.46% for GTR/SCPT2 group, 39.77 ± 21.75% for GTR/SCPT4 group). Despite the sustained elevation in NBV, there was no significant time-dependent increase from 6 to 12 weeks, indicating a plateau in bone regeneration. This suggests that the application of both EMD and SCPT peptides contributes to higher NBV levels, although the effect on bone regeneration did not show further enhancement over the observation period.

### Histological observations

**Fig. 4:**
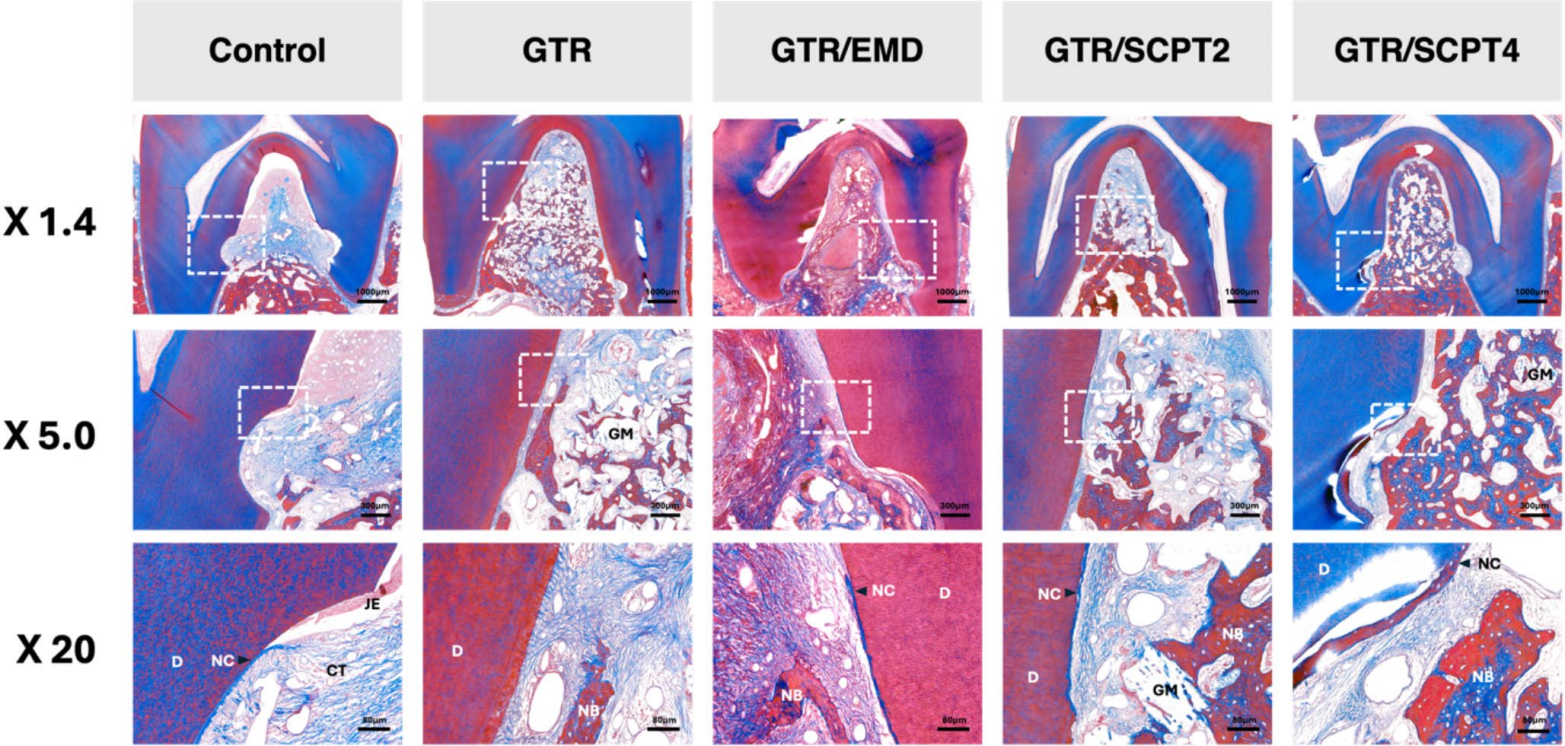
Histological analysis of periodontal defects healing at 6 weeks. Representative histological images at three different magnifications (X1.4, X5.0, X20) for each treatment group: No treatment, GTR, GTR/EMD, GTR/SCPT2, and GTR/SCPT4. The images show the healing process of periodontal defects with different treatments after 6 weeks. At the highest magnification (X 20), detailed structures such as dentin (D), new cementum (NC), connective tissue (CT), junctional epithelium (JE), periodontal ligament (PDL), and new bone (NB) are clearly visible. These histological markers indicate the level of periodontal regeneration across different treatments. Compared to the control group, the tissues in the GTR groups show a lower tendency to collapse. In the GTR/EMD, GTR/SCPT2, and GTR/SCPT4 groups, regeneration of NC (new cementum) can be observed. D: Dentin NC: New cementum CT: Connective tissue JE: Junctional epithelium PDL: Periodontal ligament NB: New bone

Histological observations at 6 weeks showed that the control group exhibited extensive epithelial downgrowth and parallel-oriented fibrous tissue adjacent to the root surface. Notably, there was tissue collapse due to the absence of a supporting scaffold material, resulting in increased tissue-free areas (Fig. 4). The GTR group exhibited substantial new bone formation and a lower tendency to collapse, with residual bone graft particles providing structural support for tissue maintenance. The GTR/EMD group displayed enhanced new cementum formation and the early stages of periodontal ligament fiber organization, including new bone formation. Both GTR/SCPT groups demonstrated regenerative patterns characterized by well-organized periodontal ligament and new bone formation around the graft particles, showing similar healing patterns regardless of the SCPT concentration.

**Fig. 5:**
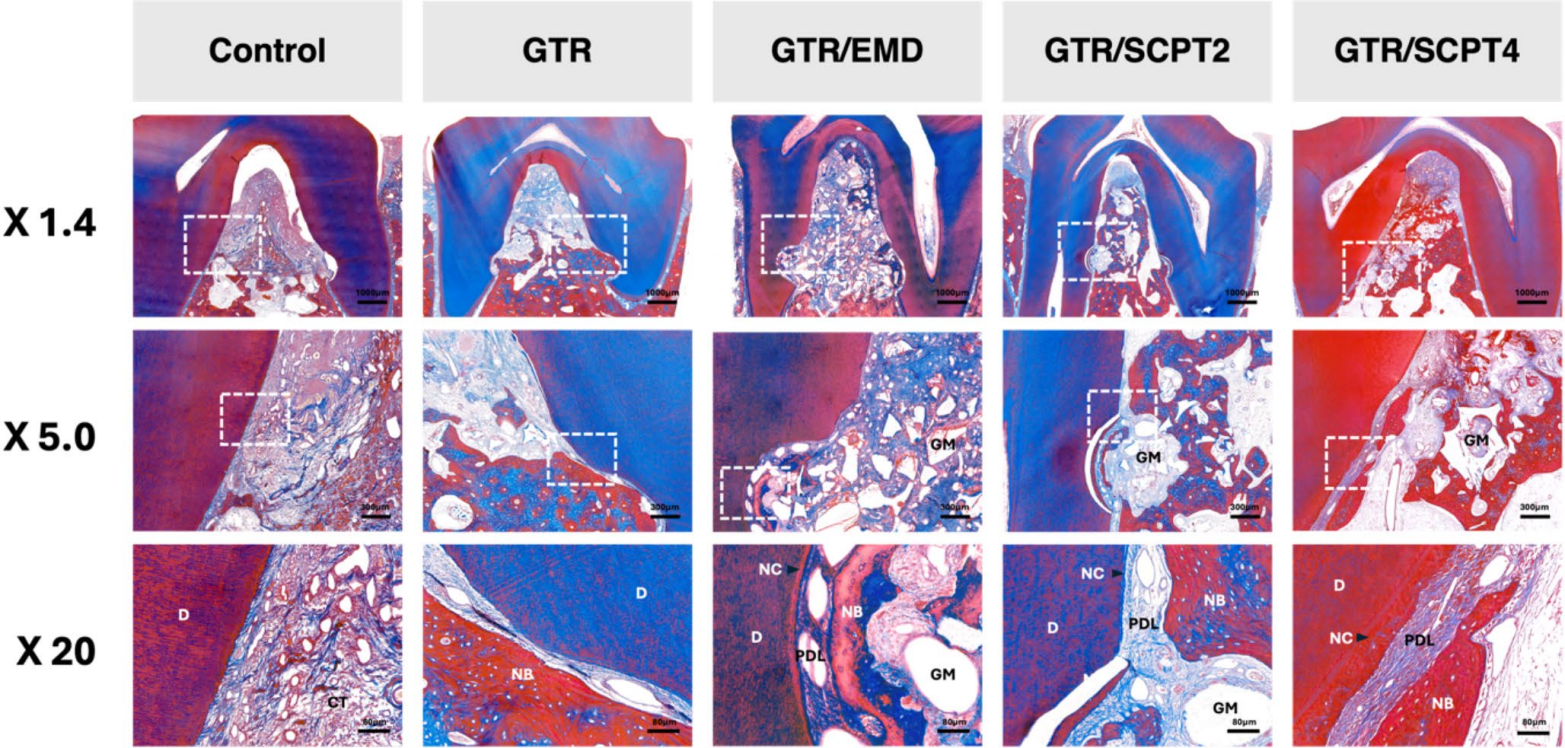
Histological analysis of periodontal defects healing at 12 weeks. Representative histological images at three different magnifications (X1.4, X5.0, X20) for each treatment group: No treatment, GTR, GTR/EMD, GTR/SCPT2, and GTR/SCPT4. The images show the healing process of periodontal defects with different treatments after 12 weeks. At the highest magnification (X20), periodontal tissue regeneration is still not well achieved in control group. In the GTR/EMD, GTR/SCPT2, and GTR/SCPT4 groups, more well-organized periodontal tissue regeneration is observed compared to the 6-week time point

By 12 weeks, the differences between the groups became more pronounced. The control group exhibited limited regeneration, characterized by loosely arranged connective tissue and compromised space maintenance, which led to tissue collapse (Fig. 5). In contrast, the GTR and GTR/EMD groups showed increased bone maturation and better organization of periodontal tissues compared to their 6-week specimens, with the GTR/EMD group displaying more pronounced cementum formation. Both concentrations of SCPT demonstrated favorable healing patterns, characterized by mature bone formation with well-defined osteocytes, dense and perpendicular-oriented periodontal ligament fibers, and continuous new cementum formation along the root surface. The bone graft particles in all treatment groups effectively maintained the space necessary for regeneration and were well-integrated within the newly formed bone tissue, showing advanced signs of bone remodeling and periodontal tissue maturation.

### Histometric analysis

#### Linear measurements

Linear measurements indicated that at both 6 and 12 weeks, all treatment groups exhibited significantly greater NCL compared to the control group (*p* < 0.0001 at 6 weeks, *p* = 0.0002 at 12 weeks) (Table 1). Notably, the high-concentration SCPT group (GTR/SCPT4) showed the highest NCL at both time points (5.47 ± 0.47 mm at 6 weeks, 5.13 ± 2.17 mm at 12 weeks). At 6 weeks, there were significant differences in CTL among the groups (*p* = 0.0005), with the control group displaying the highest values. However, these differences were not significant at 12 weeks (*p* = 0.3920).

**Table 1:** Linear measurements (mm) for each treatment modality at 6 and 12 weeks (mean ± SD; *N* = 6) abbreviations: TRL, total root length; ETL, exposed tooth length; EL, epithelial tissue length; CTL, connective tissue length; NCL, new cementum length

Time	parameters	Treatment modalities					P-value*
		(1) Control	(2) GTR	(3) GTR/EMD	(4) GTR/SCPT 2	(5) GTR/SCPT 4	
6 weeks	TRL	11.13 ± 1.84	10.38 ± 0.54	12.68 ± 2.52	12.03 ± 1.39	12.37 ± 1.41	0.2527
	ETL	1.05.±0.84	0.80 ± 1.25	0.07 ± 0.08	0.92 ± 1.39	0.23 ± 0.57	0.3131
	EL	3.40 ± 0.45	2.57 ± 1.19	2.64 ± 0.32	2.87 ± 0.42	3.04 ± 0.16	0.1761
	CTL	6.45 ± 0.37 ^a)^	4.99 ± 0.98 ^a), b)^	4.13 ± 1.17 ^b)^	4.31 ± 1.11 ^b)^	3.78 ± 0.97 ^b)^	0.0005
	NCL	0.48 ± 0.11 ^a)^	2.58 ± 0.58 ^a), b)^	4.60 ± 1.16 ^b)^	3.68 ± 0.74 ^b)^	5.47 ± 0.47 ^b)^	< 0.0001
12 weeks	TRL	10.96 ± 1.25	10.38 ± 0.54	12.68 ± 2.52	10.03 ± 5.01	12.37 ± 1.41	0.4118
	ETL	2.71 ± 1.08	0.13 ± 0.26	0.52 ± 1.28	1.65 ± 2.78	0.34 ± 0.53	0.0357
	EL	2.88 ± 0.44	3.24 ± 0.36	3.28 ± 0.45	3.03 ± 0.28	3.36 ± 0.25	0.1898
	CTL	4.36 ± 1.79	3.17 ± 1.03	4.09 ± 1.29	2.84 ± 1.73	3.54 ± 0.89	0.3920
	NCL	1.02 ± 0.24 ^a)^	3.85 ± 0.43 ^b)^	4.79 ± 1.60 ^b)^	4.52 ± 0.90 ^b)^	5.13 ± 2.17 ^b)^	0.0002

* P-value by one-way ANOVA

Different letters, a, and b, indicate statistical significance under Tukey’s multiple comparison test

#### Area measurements

The area measurements indicated that all treatment groups exhibited significantly larger HTA than the control group at both 6 and 12 weeks (*p* < 0.0001 and *p* = 0.0018, respectively) (Table 2). Although not statistically significant, the groups treated with SCPT tended to have higher NBA values than those treated with GTR or GTR/EMD. Notably, the high-concentration SCPT group (GTR/SCPT4) demonstrated the highest values in NBA formation among all groups at both time points, recording 40.83 ± 5.90% at 6 weeks and 43.35 ± 16.56% at 12 weeks, with these values being statistically significant compared to the control group (*p* = 0.0171 at 6 weeks, *p* = 0.0047 at 12 weeks). By 12 weeks, EA and CA were significantly reduced in both SCPT groups and the EMD group relative to the control group (*p* = 0.0035 and *p* = 0.0045, respectively). There were no significant differences in the RGA and NFA among the treatment groups at either time point (*p* = 0.6015 at 6 weeks, *p* = 0.6395 at 12 weeks).

**Table 2:** Area measurements (%) for each treatment modality at 6 and 12 weeks (mean ± SD; *N* = 6)

Time	parameters	Treatment modalities					P-value*
		(1) Control	(2) GTR	(3) GTR/EMD	(4) GTR/SCPT 2	(5) GTR/SCPT 4	
6 weeks	NFA	7.56 ± 14.24	0.57 ± 0.89	0.43 ± 0.50	0.12 ± 0.19	0.07 ± 0.17	0.4117
	EA	8.01 ± 10.29	2.69 ± 1.60	1.90 ± 1.24	1.55 ± 2.27	1.53 ± 3.74	0.1649
	NBA	20.60 ± 10.86 ^a)^	26.69 ± 11.39 ^a), b)^	35.61 ± 12.85 ^a), b)^	37.35 ± 10.81 ^a), b)^	40.83 ± 5.90 ^b)^	0.0171
	RGA	-	19.26 ± 9.63	18.46 ± 5.55	15.85 ± 1.63	15.13 ± 4.91	0.6015
	CA	59.50 ± 14.72	50.80 ± 11.70	43.59 ± 8.82	45.12 ± 12.38	42.44 ± 7.04	0.0892
	HTA	20.60 ± 10.86^a)^	45.94 ± 12.44 ^b)^	54.08 ± 10.30 ^b)^	53.21 ± 10.69 ^b)^	55.96 ± 7.16 ^b)^	< 0.0001
12 weeks	NFA	7.36 ± 6.76	3.11 ± 6.22	0.42 ± 1.03	3.86 ± 6.55	2.66 ± 4.93	0.3009
	EA	10.59 ± 2.39 ^a)^	5.42 ± 4.57 ^a), b)^	1.13 ± 1.96 ^b)^	4.94 ± 7.27 ^a), b)^	1.58 ± 2.45 ^b)^	0.0035
	NBA	12.85 ± 5.17 ^a)^	31.77 ± 12.42 ^a), b)^	30.45 ± 10.89 ^a), b)^	40.00 ± 15.83 ^b)^	43.35 ± 16.56 ^b)^	0.0047
	RGA	-	15.52 ± 3.66	16.71 ± 4.89	11.68 ± 9.22	13.58 ± 7.32	0.6395
	CA	69.20 ± 8.99 ^a)^	44.18 ± 5.82 ^a), b)^	51.30 ± 12.34 ^a), b)^	39.51 ± 16.54 ^b)^	38.83 ± 17.59 ^b)^	0.0045
	HTA	12.85 ± 5.17 ^a)^	47.29 ± 15.46 ^b)^	47.16 ± 13.45 ^b)^	51.69 ± 23.91 ^b)^	56.93 ± 21.76 ^b)^	0.0018

Abbreviations: NFA, non-filled area; EA, epithelial tissue area; NBA, new bone area; RGA, residual graft area; CA, connective tissue area; HTA, hard tissue area including NBA and RGA

* P-value by one-way ANOVA

Different letters, a, and b, indicate statistical significance under Tukey’s multiple comparison test

## Discussion

This study aimed to evaluate the regenerative potential of the SCPT peptide in a class II furcation defect model. Using a combination of GTR and bioactive agents, including EMD and two concentrations of SCPT, periodontal regeneration was assessed through micro-CT, histologic, and histometric analyses at 6 and 12 weeks. The findings demonstrated significant improvements in NBV in groups treated with SCPT compared to the control, with no additional NBV gains at increased SCPT concentrations. Furthermore, histologic and histometric observations indicated superior structural organization and cementum formation in treated groups, suggesting that SCPT holds potential as a bioactive agent for periodontal regeneration.

Radiographic, histologic, and histometric analyses conducted at 6 and 12 weeks demonstrated significant differences in bone volume and structural organization among the treatment groups. The collagenated block type bone substitute used in GTR served as a scaffold to prevent tissue collapse, resulting in enhanced NBV, NBA, and HTA compared to the control group. In a clinical study, collagenated soft block bone substitutes yielded favorable clinical and radiographic outcomes for guided tissue regeneration treatment in class II furcation defects over a 1-year follow-up period [3]. These findings suggest that the use of scaffolds for space provision and maintenance plays a vital role in periodontal tissue regeneration.

Another important aspect of regeneration in GTR procedures may be cell exclusivity, which is achieved through the use of a barrier membrane [10]. However, studies on regenerative treatment with EMD have shown limited evidence supporting the benefits of using a barrier membrane in combination with EMD [33]. Several studies have reported the formation of new attachment using EMD alone in treatment of intrabony and furcation defects [34, 35]. Despite such clinical evidence, this study utilized a barrier membrane in combination with EMD or SCPT in all test groups. Because the GTR group, which served as a positive control, utilized a bone substitute combined with a barrier membrane, all other test groups used a barrier membrane as well to eliminate confounding variables and solely compare the additive effect of the biologic agents.

Histological examination revealed that specimens treated with SCPT displayed enhanced organization of the periodontal ligament and cementum regeneration, similar to those treated with EMD. The SCPT-treated groups also showed improved collagen fiber orientation and maturation, suggesting that SCPT facilitates the organization of the extracellular matrix in a manner comparable to well-known bioactive molecules such as EMD [36, 37] and PDGF [38, 39] in periodontal regeneration procedures. These findings support earlier histological studies that link SCPT administration to cytoskeletal rearrangement, which is essential for maintaining ligament structural integrity and forming the attachment apparatus [30]. Additionally, SCPT-treated specimens demonstrated minimal epithelial downgrowth, a crucial factor in differentiating regenerative from reparative healing outcomes, as indicated in previous research. Previous studies on SCPT applications have consistently shown a modulation of cellular responses that favors regeneration [26, 30]. Although EMD is known for its effectiveness in inhibiting epithelial migration [40], the similar therapeutic results observed with SCPT warrant further comparative studies.

The adjunctive use of EMD or two concentrations of SCPT in GTR resulted in higher values of NBV, NCL, NBA, and HTA compared to GTR alone, although these differences were not statistically significant at 6 and 12 weeks. Previous studies have shown that the adjunctive use of EMD or SCPT with bone substitutes significantly enhances periodontal regeneration compared to bone grafting alone in the treatment of infrabony defects [30, 41]. Unlike these earlier findings, our current study did not find significant additional benefits of EMD and SCPT compared to the GTR alone group. However, it is important to note that the use of bioactive agents such as EMD and SCPT did show statistically significant improvements compared to the control group in certain parameters where the GTR alone group did not, specifically in NBV at 6 and 12 weeks, NCL at 6 weeks, and NBA at 6 and 12 weeks.

The absence of a time-dependent increase in NBV from 6 to 12 weeks suggests that bone regeneration reached a plateau, indicating no further benefits over time. This plateau effect emphasizes the initial effectiveness of SCPT in promoting bone formation, while also suggesting a possible saturation in its regenerative capacity. Additionally, the structural support provided by bone graft particles in these groups seemed crucial for maintaining space, which assists in the maturation of regenerated bone. These findings highlight the potential of SCPT as a bioactive agent for periodontal regeneration, providing a consistent increase in bone volume comparable to established agents such as EMD.

At 6- and 12-week observation periods, the SCPT-treated groups exhibited well-organized periodontal structures, with mature bone formation and densely aligned periodontal ligament fibers. Despite the high concentration of SCPT, there were no adverse effects on bone formation, unlike other agents such as rhBMP-2, which may cause complications at elevated doses. Previous studies have shown that suboptimal concentrations of rhBMP-2 can lead to compromised bone mineralization, disruption of osteoclast-osteoblast homeostasis, and adverse soft tissue manifestations, including facial edema [41]. In our previous investigation, a low concentration of rhBMP-2 (0.2 mg/ml) demonstrated enhanced periodontal tissue regeneration compared to the control group, as evidenced by reduced defect height and increased formation of new cementum and bone [12]. However, a higher concentration (0.5 mg/ml) exhibited diminished regenerative effects on periodontal tissues [12]. Furthermore, some specimens treated with higher concentrations exhibited ankylosis. These findings underscore the critical importance of examining not only the therapeutic efficacy, but also the safety threshold, when utilizing bioactive agents at elevated concentrations. In the present study, histological specimens from the high-concentration SCPT administration group showed no adverse effects, such as ankylosis, inflammatory responses, or bone resorption. This safety profile at high doses suggests that SCPT could be a viable alternative to traditional regenerative materials in complex defects requiring bioactivity and structural stability. Histological analysis further confirmed the improved formation of new cementum in SCPT groups, supporting its role in facilitating the attachment of periodontal ligament cells. Collectively, the radiographic and histologic findings validate SCPT’s regenerative efficacy, encouraging further exploration in clinical applications with a high safety margin up to a concentration of 4 mg/ml.

One limitation of this study was that the EMD and SCPT have a semi-fluid nature that may have allowed the materials to permeate into adjacent sites. The proximity of the defects may have also allowed co-contamination of the sites by the different test groups. Additionally, the observation period was restricted to 12 weeks, highlighting the need for further evaluation of SCPT’s long-term stability and functional performance. Another limitation is the absence of a detailed analysis of vascular changes in the regenerating tissue, which could shed light on the plateau effect observed in periodontal regeneration. Future research should consider extended observation periods and alternative administration methods to maximize the clinical potential of SCPT.

## Conclusion

In conclusion, SCPT demonstrated promising regenerative capabilities in periodontal tissue, yielding outcomes comparable to those of EMD in terms of NBV, PDL organization, and new cementum formation. Both the EMD and SCPT groups outperformed the controls, highlighting SCPT’s potential as an alternative bioactive agent for periodontal regeneration. Future studies that focus on long-term efficacy and optimizing SCPT delivery could pave the way for improved therapeutic protocols in the treatment of complex periodontal defects.

## Data Availability

The data supporting this study’s findings are available upon request from the corresponding authors.
